# Supplementary material for: Large linear non-saturating magnetoresistance and high mobility in ferromagnetic MnBi
Source: Nat Commun. 2021 Jul 28;12:4576. doi: 10.1038/s41467-021-24692-7 (PMC8319177; doi:10.1038/s41467-021-24692-7)
Supplement: Supplementary file 1 — Supplementary Information [file 41467_2021_24692_MOESM1_ESM.pdf]

# Large linear non-saturating magnetoresistance and high mobility in ferromagnetic topological material MnBi

## Supplemental information

Yangkun He[1*], Jacob Gayles[1,2], Mengyu Yao[1], Toni Helm[1,3], Tommy Reimann[3], Vladimir N. Strocov[4], Walter Schnelle[1], Michael Nicklas[1], Yan Sun[1], Gerhard H. Fecher[1], Claudia Felser[1]

[1]*Max-Planck-Institute for Chemical Physics of Solids, D-01187 Dresden, Germany*

[2]*Department of Physics, University of South Florida, Tampa, Florida 33620, USA*

[3]*Dresden High Magnetic Field Laboratory (HLD-EMFL), Helmholtz-Zentrum Dresden–Rossendorf, 01328 Dresden, Germany*

[4]*Swiss Light Source, Paul Scherrer Institut, CH-5232 Villigen, Switzerland*

*email: yangkun.he@cpfs.mpg.de

## 1. Crystal structure

The crystal structure was characterised using powder X-ray diffraction at room temperature, and the results are summarised in Supplemental Table 1.

**Supplemental Table 1. Crystal structure of MnBi at room temperature**

| | Lattice constant | | | | | Sites and occupation | |
|---|---|---|---|---|---|---|---|
| Space group | *a* | *c* | *c*/*a* | *V* (f.u.) | Density | Mn | Bi |
| $P6_3$/m m c | 4.2876(5) Å | 6.1154(5) Å | 1.4263 | 48.68(0) $Å^3$ | 9.00 g/$cm^3$ | (0 0 0) | (1/3 2/3 1/4) |
| 194 | | | | | | 1 | 1 |

## 2. Residual-resistance ratio (RRR)

MnBi exhibits metallic behaviour with a large residual resistivity ratio (RRR) of 100 along the *a*-axis and 40 along the *c*-axis, which indicates the high quality of the single crystal and a very high mobility, which is suppressed by phonon scattering during heating. The RRR of other ferromagnetic topological materials is shown for a comparison. Generally, their RRR is low owing to the low mobility caused by the magnetic disorder scattering and atomic disorder (e.g. in $Co_2MnGa$).

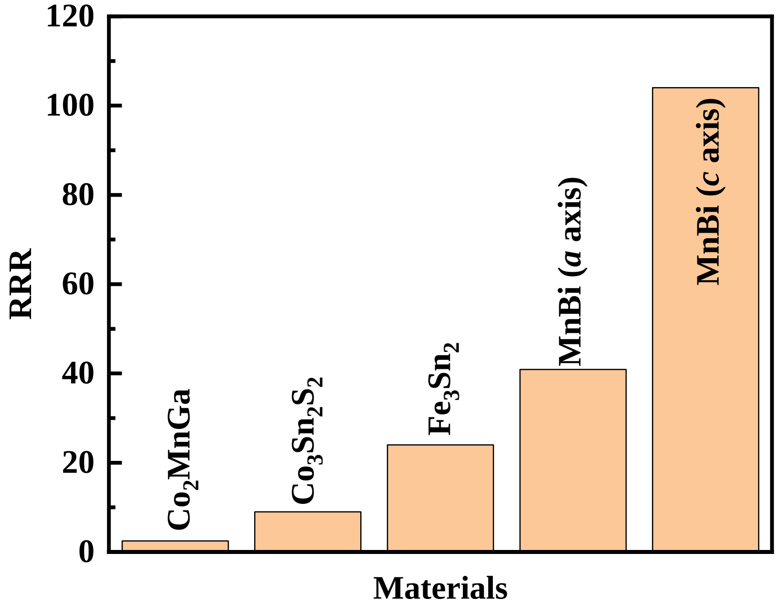


**Supplementary Figure 1. RRR of ferromagnetic materials.** Data of $Fe_3Sn_2$, $Co_3Sn_2S_2$ and $Co_2MnGa$ are from Refs. 1-3.

## 3. Oscillation in magnetoresistivity

The magnetoresistance (MR) was measured at a low temperature with a magnetic field along the *a*-axis and the current along the *c*-axis. Supplementary Fig. 2a shows the data measured in a direct current (DC) field at 1 K. The saturation magnetic field is 0.5 T. The extracted amplitudes of the SdH oscillations as a function of the inverse

magnetic field ($1/B$) by subtracting a cubic polynomial from the resistivity data below 14 T are shown in Supplementary Fig. 2c. The fast Fourier transform (FFT) of the SdH oscillations shows a dominant single frequency of 23 T in Supplementary Fig. 2d. The data measured in the pulsed field are presented in Supplementary Fig. 2b. The plot is too noisy to distinguish the oscillation from the noise in the pulsed field, but a linearly increasing MR of 5000% can be observed.

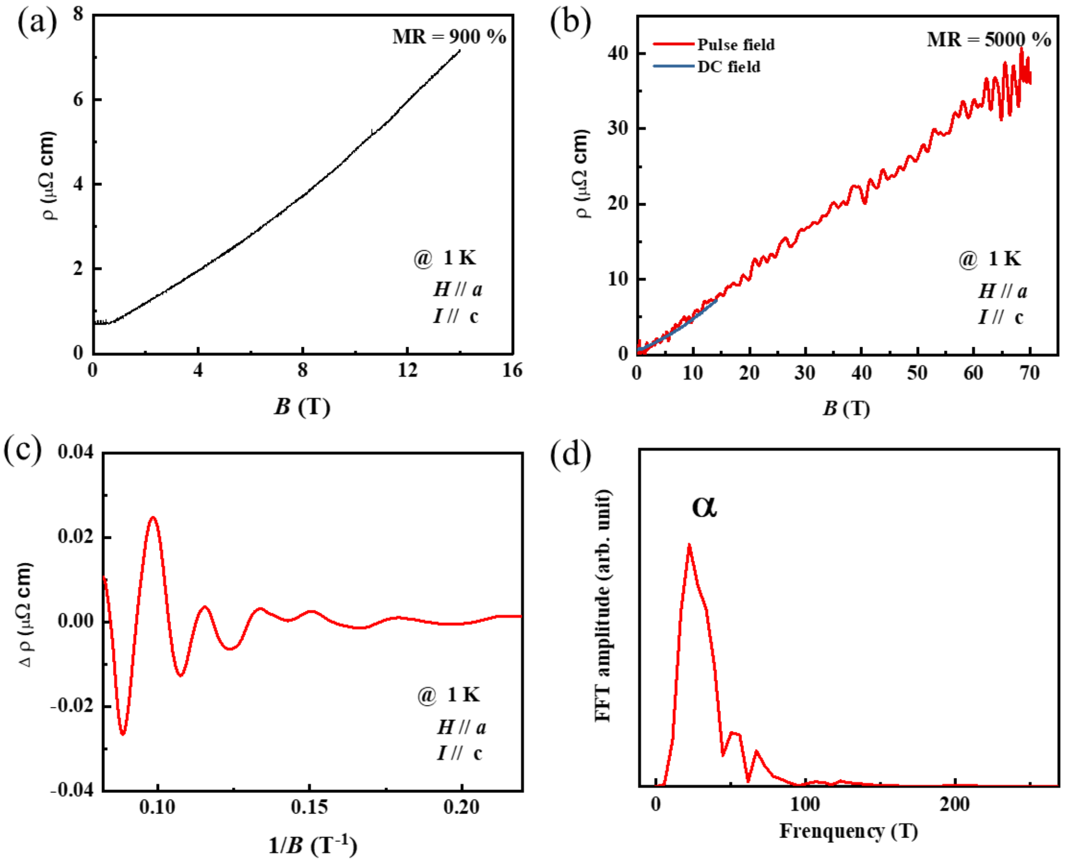


**Supplementary Figure 2. Oscillation in the longitudinal MR.** (a) MR measured under an applied DC field at 1 K. (b) MR measured under a pulse field at 1 K up to 70 T, showing a linear increase. The DC data are also shown for a comparison. (c) Extracted amplitudes of the SdH oscillations as a function of the inverse magnetic field ($1/B$) using the DC data below 14 T. (d) FFT of the SdH oscillations at 1 K.

## 4. Anomalous Hall and topological Hall effects of MnBi when *H*//*a* and *I*//*c*

The Hall resistivity $\rho_H$ was measured when the field was parallel to the *a*-axis, and the current was along the *c*-axis. The intrinsic anomalous Hall conductivity, deduced from a linear fit of $\rho_H$ versus the square of the longitudinal resistivity $\rho^2$, is -450 $\Omega^{-1}$ $cm^{-1}$.

Despite the anomalous Hall effect, the topological Hall effect, namely an additional bump during magnetisation, is shown above 87 K. The topological Hall effect is due to the non-coplanar easy-cone structure above the spin-reorientation temperature $T_{SR2}$. The topological Hall resistivity $\rho_{THE}$ is calculated using the following

equation for $\rho_H$:

$$\rho_H = R_0 B + R_S \mu_0 M + \rho_{THE} \tag{1}$$

where $R_0$ and $R_S$ are the ordinary and anomalous Hall coefficients, respectively, $B$ is the magnetic field, $\mu_0$ is the permeability of free space, and $M$ is the magnetisation. The topological Hall conductivity $\sigma_{THE}$ (calculated by $\sigma_{THE} = -\rho_{THE}/\rho^2$) versus temperature curve (Supplementary Fig. 3f) shows a peak at approximately 130 K in the easy-cone magnetic structure, which is an intermediate state between the easy plane and the easy axis structures.

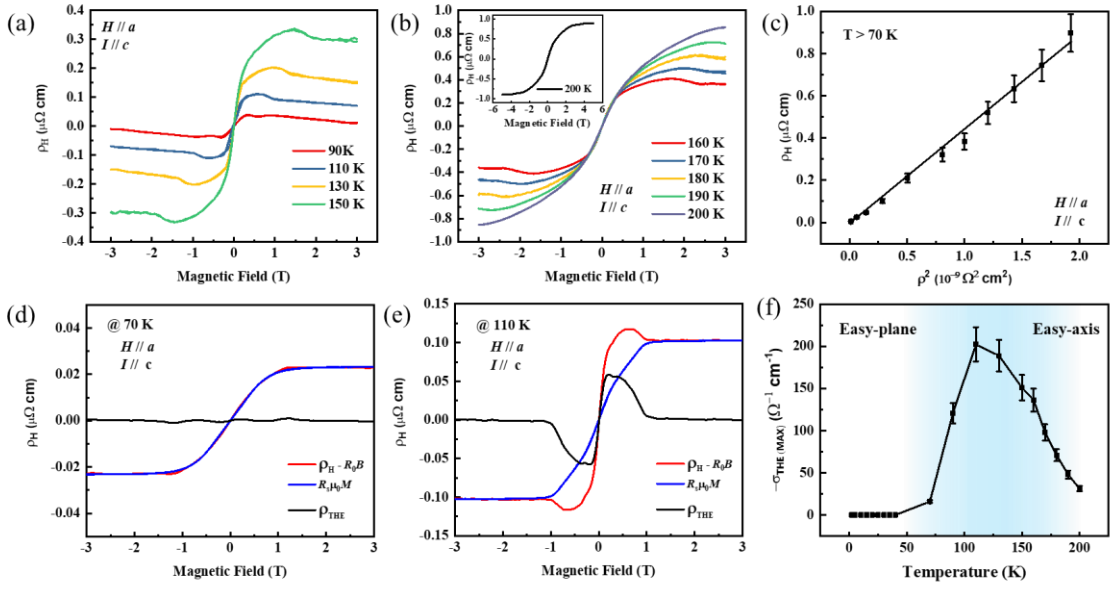


**Supplementary Figure 3. Anomalous Hall and topological Hall effects when *H*//*a* and *I*//*c*.** (a) and (b) Hall resistivity at different temperatures. (c) $\rho_H$ versus $\rho^2$. (d) and (e) Fitting the topological Hall effect at different temperatures. At 70 K, the topological Hall resistivity is zero, whereas a topological Hall resistivity of 0.05 μΩ cm is observed at 110 K when the magnetic structure prefers an easy cone. (f) Topological Hall conductivity at different temperatures. The error bar due to the uncertainty of the geometry of the sample is estimated as 10%.

## 5. Fermi surface

The fermi surface of MnBi is shown in Supplementary Fig. 4. There are mainly five bands crossing the Fermi level, with three typical hole pockets around Γ point (see Supplementary Fig. 4a-4c) and two typical electron pockets locating at M and K points, respectively (see Supplementary Fig. 4e). These bands are mainly from Bi 6*p* band. Band in Supplementary Fig. 4d comes from the strongly hybridised Mn 3*d* and Bi 6*p* band, which contribute to most of the charge carrier density.

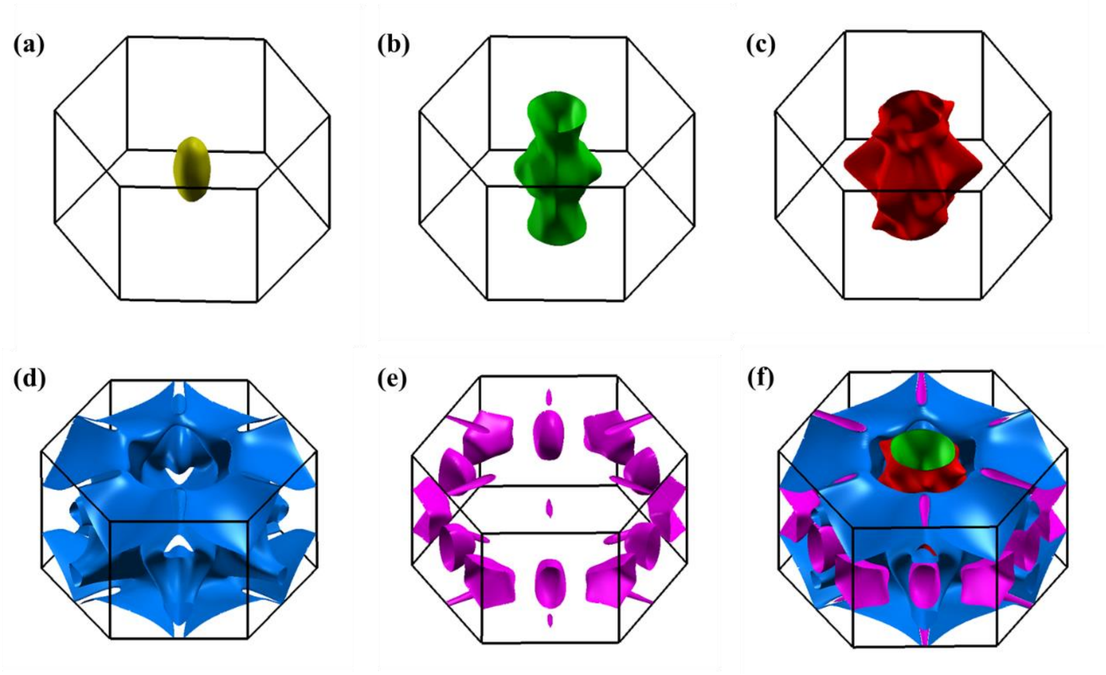


**Supplementary Figure 4. MnBi Fermi surface.** (a)-(e) show individual Fermi surfaces of each band. (f) is the combination of all bands. There are mainly five bands crossing the Fermi level.

## 6. Topological nature of MnBi

The band near the Fermi level on the cut between $M_2$ and $M_3$ is dominated by the spin up channel, as presented in Supplementary Fig. 5. A band inversion occurs at 0.25 eV below the Fermi level. Because of the mirror symmetry $\{m_{001}|0, 0, 1/2\}$, the bands in the mirror symmetry invariant plane with $k_z$=0 can have the mirror eigenvalues 1 and -1. If two inverted bands have opposite mirror eigenvalues, a nodal line linear crossing can be formed in the $k_z$=0 plane. Indeed, the two linear crossing points between $M_2$ and $M_3$ are just locating on such nodal lines. The mirror symmetry can be broken by the spin-orbit coupling (SOC) and the applied magnetic field (not perpendicular to the mirror plane). The nodal line linear band crossing can be broken by opening a bandgap with one pair of Weyl points locating on the original nodal lines, similar to the case in $Co_3Sn_2S_2$[1]. This bandgap can have strong local Berry curvature around the mirror plane and leads to a strong anomalous Hall effect.

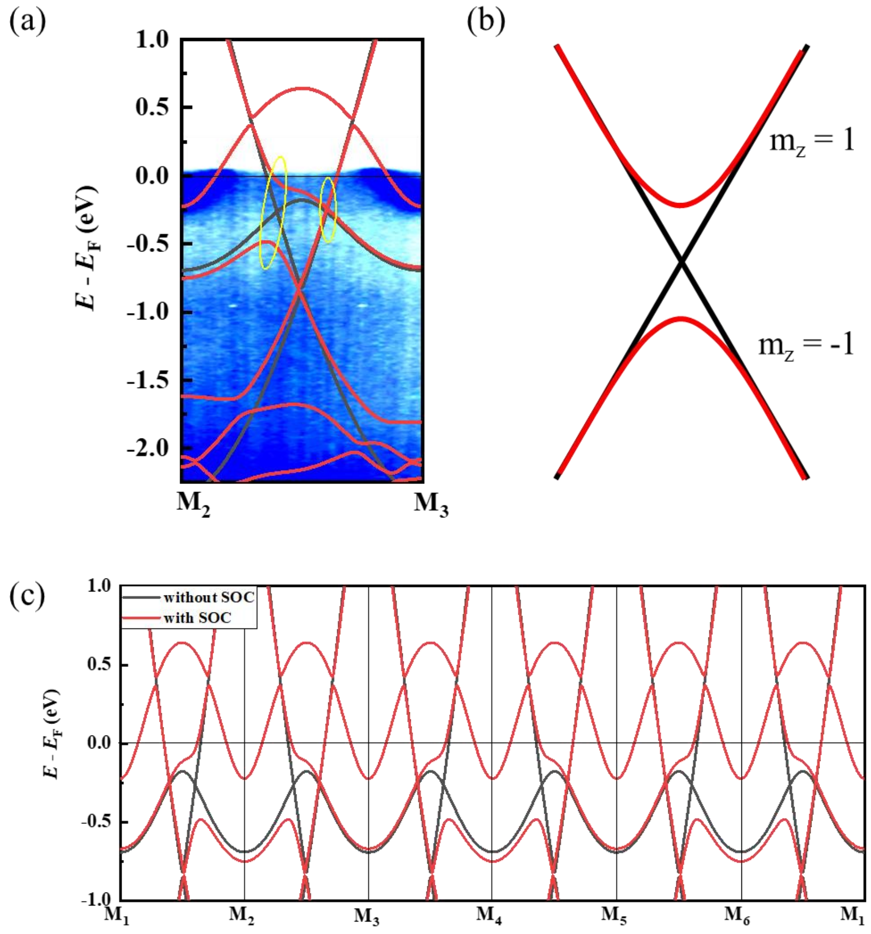


**Supplementary Figure 5. Topological nature of MnBi.** (a) Calculated band structure with ARPES experiment. The red and black lines are corresponding to bands with and without SOC, respectively. Nodal lines and Weyl points are marked in yellow circles. (b) Schematical view for the band inversion. (c) Calculated band structure with the moment along (100) direction. Magnetic moment breaks the six-fold symmetry, forming nodal lines and Weyl points depending on the moment direction. It is a pity that MnBi has easy-plane magnetic structure at low temperature, so that the ARPES result is an average of hundreds of magnetic domains with different magnetization directions. Therefore, it is difficult to observe clearly nodal lines and Weyl points by experiment.

## 7. Fermi surface map in the $k_x$–$k_z$ plane at $k_y = 0$

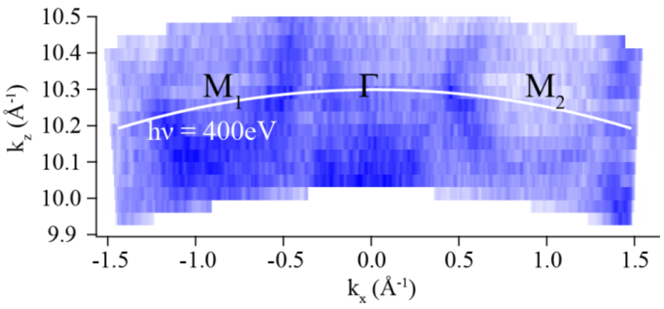


**Supplementary Figure 6. ARPES intensity plot in the $k_x$–$k_z$ plane at $k_y = 0$, 48 meV**

**below $E_F$.** The figure was acquired with circular polarized photon with photon energy ranging from 380 to 420 eV. The $M_1$-Γ-$M_2$ plane lies at $k_z$ = 10.27 Å. The hole pocket at Γ point and electron pocket at M points can be clearly seen.

## 8. Fermi velocity and effective mass

The observed electron bands were fitted on their energy-distribution curves (EDCs) with a parabola to calculate the Fermi velocity $V_F$ and effective mass. The results of the fits are as follows: These bands are responsible for the low-frequency oscillations shown in Supplementary Fig. 2.

Band 1 (from $M_1$–Γ direction): $V_F$ = 3.6 eVÅ, $m$ = 2.54, $m_0$ ($m_0$ is the mass of an electron)
Band 2: $V_F$ = 7.3 eVÅ, $m$ = -0.85 $m_0$
Band 1' (from $M_1$–$M_2$ direction): $V_F$ = 10.2 eVÅ, $m$ = 0.42 $m_0$

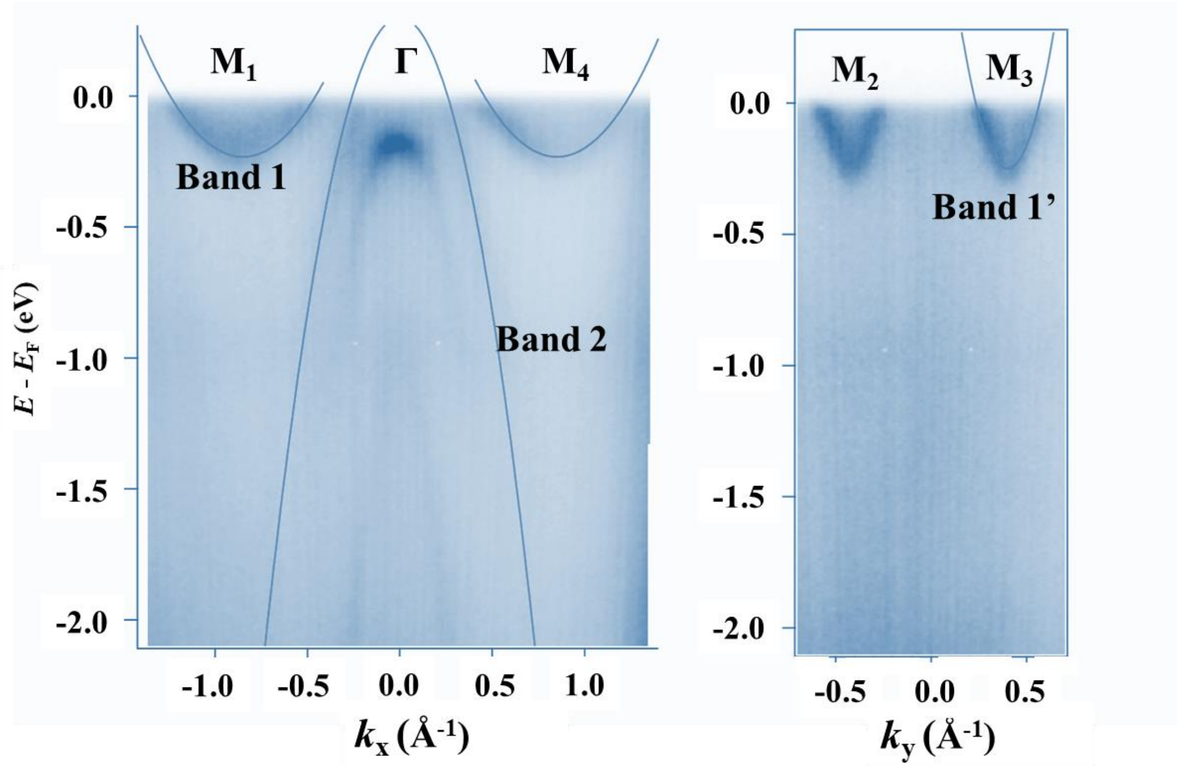


**Supplementary Figure 7. Band structure fitted with parabolas.**

## References


1. Liu, E., Sun, Y., Kumar, N. et al. Giant anomalous Hall effect in a ferromagnetic kagome-lattice semimetal. Nat. Phys. **14**, 1125–1131 (2018).

2. Ye, L., Kang, M., Liu, J. et al. Massive Dirac fermions in a ferromagnetic kagome metal. Nature **555**, 638–642 (2018).

3. Manna, K., Muechler, L., & Kao, T., From colossal to zero: Controlling the anomalous Hall effect in magnetic Heusler compounds via Berry curvature design, Phys. Rev. X **8**, 041045 (2018).
